# Supplementary material for: Reconciling Mining with the Conservation of Cave Biodiversity: A Quantitative Baseline to Help Establish Conservation Priorities
Source: PLoS One. 2016 Dec 20;11(12):e0168348. doi: 10.1371/journal.pone.0168348 (PMC5173368; doi:10.1371/journal.pone.0168348)
Supplement: S1 Dataset — (ZIP) [file pone.0168348.s002.zip › Taxa/Serra Norte/SN_2013/N5S/Lista N5S-79.pdf]

CAVIDADE N5S-0079

Classe	Ordem	Fam/Outros	Gên/Outros	Espécie	S-U	U-S	Zon.
Arachnida	Acari	Anystidae	*Erythracarus*	sp.1	X		E/P
Arachnida	Amblypygi	Phrynidae	*Heterophrynus*	*longicornis*	4	6	E/P
Arachnida	Araneae	Araneidae		jovem		X	E/P
Arachnida	Araneae	Caponiidae		jovem	X	X	P
Arachnida	Araneae	Corinnidae		jovem	1		P
Arachnida	Araneae	Ctenidae		jovem	2	1	P
Arachnida	Araneae	Drymusidae	*Drymusa*	*spelunca*	X	X	E/P
Arachnida	Araneae	Mysmenidae	*Microdipoena*	sp.1		X	E/P
Arachnida	Araneae	Ochyroceratidae	*Ochyrocera*	sp.3		X	E/P
Arachnida	Araneae	Ochyroceratidae	*Speocera*	sp.1		X	E/P
Arachnida	Araneae	Ochyroceratidae		jovem	X	X	E/P
Arachnida	Araneae	Pholcidae	*Mesabolivar*	*aurantiacus*	X	X	E/P
Arachnida	Araneae	Pholcidae	*Mesabolivar*	sp.1		X	E/P
Arachnida	Araneae	Pisauridae		jovem	2	2	E/P
Arachnida	Araneae	Salticidae		sp.14		X	E
Arachnida	Araneae	Theridiidae	*Theridion*	sp.2	X	X	E/P
Arachnida	Araneae	Theridiidae	*Theridion*	sp.4	X	X	P
Arachnida	Araneae	Theridiosomatidae	*Plato*	sp.1	X		E/P
Arachnida	Araneae	Theridiosomatidae	*Natlo*	sp.1	X		E/P
Arachnida	Araneae	Theridiosomatidae	*Plato*	sp.1	X	X	E/P
Arachnida	Opiliones	Escadabiidae		sp.2		X	P
Arachnida	Opiliones	Stygnidae	*Protimesius*	*gracilis*	1		E/P
Arachnida	Pseudoscorpiones	Chthoniidae	*Pseudochthonius*	sp.1		X	E/P
Arachnida	Schizomida	Hubbardiidae		jovem	X		E/P
Chilopoda	Geophilomorpha	Geophilidae		sp.1	1		E/P
Chilopoda	Scolopendromorpha	Scolopendridae	*Otostigmus*	sp.1	2		P
Chilopoda	Scolopendromorpha	Scolopocryptopidae	*Newportia*	sp.1	1		E
Chilopoda	Scutigeromorpha	Pselliodidae	*Sphendononema*	jovem	1		E
Diplopoda	Polydesmida	Chelodesmidae		jovem		2	E/P
Diplopoda	Polydesmida	Fuhrmannodesmidae		jovem		X	P
Diplopoda	Polydesmida	Pyrgodesmidae		jovem	X	X	P
Diplopoda	Siphonophorida	Siphonophoridae		jovem		X	P
Entognatha	Collembola	Cyphoderidae		sp.1		X	E/P
Entognatha	Collembola	Paronellidae		sp.1	X	X	E/P
Entognatha	Diplura	Campodeidae		sp.1		X	E/P
Insecta	Blattodea	Polyphagidae		sp.1	5	1	E/P
Insecta	Coleoptera	Carabidae		sp.3		X	P
Insecta	Coleoptera	Coccinellidae		sp.1		X	E
Insecta	Coleoptera	Curculionidae	Scolytinae	sp.2	X		E/P
Insecta	Coleoptera	Scydmaenidae		sp.6		X	E
Insecta	Coleoptera	Staphylinidae	Pselaphinae	sp.4		X	P
Insecta	Coleoptera	Staphylinidae	Pselaphinae	sp.5	X		E/P
Insecta	Coleoptera	Staphylinidae		sp.4		X	P
Insecta	Diptera	Cecidomyiidae	Cecidomyiinae	sp.	X	X	E/P
Insecta	Diptera	Ceratopogonidae		sp.	X		P
Insecta	Diptera	Chaoboridae		sp.		X	E/P
Insecta	Diptera	Culicidae	*Culex*	sp.		X	E/P
Insecta	Diptera	Psychodidae	Phlebotominae	sp.		X	P
Insecta	Diptera	Tipulidae	Tipulinae	sp.	X	X	E/P

Insecta	Heteroptera	Dipsocoroidea		sp.	X		E/P
Insecta	Heteroptera	Reduviidae	Reduviinae	sp.	1	1	E/P
Insecta	Heteroptera	Veliidae	*Microvelia*	sp.	X	X	E
Insecta	Homoptera	Cixiidae		jovem	X	X	E/P
Insecta	Hymenoptera	Formicidae	*Camponotus*	sp.2	X	X	e
Insecta	Hymenoptera	Formicidae	*Crematogaster*	sp.1		X	E/P
Insecta	Hymenoptera	Formicidae	*Gnamptogenys*	sp.1	X	X	E/P
Insecta	Hymenoptera	Formicidae	*Hypoponera*	sp.1		X	E/P
Insecta	Hymenoptera	Formicidae	*Odontomachus*	sp.1	X		E
Insecta	Hymenoptera	Formicidae	*Paratrechina*	sp.1	X	X	E/P
Insecta	Hymenoptera	Formicidae	*Paratrechina*	sp.2	X	X	E/P
Insecta	Hymenoptera	Formicidae	*Pheidole*	sp.2	X		E/P
Insecta	Isoptera	Termitidae	*Nasutitermes*	sp.		X	E/P
Insecta	Lepidoptera	Noctuoidea		sp.6		1	E/P
Insecta	Lepidoptera	Tineoidea		sp.2	X	X	P
Insecta	Orthoptera	Gryllidae	Nemobiinae	jovem		1	E/P
Insecta	Orthoptera	Phalangopsidae	*Paraclodes*	sp.	8	5	E/P
Insecta	Orthoptera	Phalangopsidae	*Phalangopsis*	sp.	14	40	P
Insecta	Psocoptera	Epipsocidae	*Epipsocus*	sp.2	X		P
Insecta	Thysanura	Nicoletiidae		sp.1	X		P
Gastropoda	Pulmonata	Systrophiidae	*Happia*	sp.	X		E
Malacostraca	Isopoda	Dubioniscidae		sp.1	X		E/P
Malacostraca	Isopoda	Philosciidae		sp.1	X	X	E/P
Malacostraca	Isopoda	Philosciidae		sp.2	X		E
Amphibia	Anura			(girinos)		30	E
Amphibia	Anura			sp.	3	1	E/P
Mammalia	Chiroptera	Emballonuridae	*Peropteryx*	*macrotis*		4	E
Mammalia	Chiroptera	Furipteridae	*Furipterus*	*horrens*	1		P
